# Measurement of motor-evoked potential resting threshold and amplitude of proximal and distal arm muscles in healthy adults. A reliability study

**DOI:** 10.1177/2055668318765406

**Published:** 2018-04-02

**Authors:** Lisa Tedesco Triccas, Ann-Marie Hughes, Jane H Burridge, Amy E Din, Martin Warner, Simon Brown, Mahalekeshmi Desikan, John Rothwell, Geert Verheyden

**Affiliations:** 1 Faculty of Health Sciences, 34584 University of Southampton, Southampton, UK; 2 Department of Rehabilitation Sciences, KU Leuven, Leuven, Belgium; 3 Institute of Neurology, Queen Square, University College London, London, UK

**Keywords:** Transcranial magnetic stimulation, reliability, upper limb, proximal upper limb muscles, distal upper limb muscles, outcome measurement, neurorehabilitation, minimal detectable change

## Abstract

**Purpose:**

Reliability of motor-evoked potential threshold and amplitude measurement of upper limb muscles is important when detecting changes in cortical excitability. The objective of this study was to investigate intra-rater, test–retest reliability and minimal detectable change of resting motor threshold and amplitude of a proximal and distal upper limb muscles, anterior deltoid and distal extensor digitorum communis in healthy adults.

**Method:**

To measure motor-evoked potential responses, transcranial magnetic stimulation was interfaced with electromyography and neuronavigation equipment. Two measurements were conducted on day 1 and a third measurement three days later. Reliability was analysed using intraclass correlation coefficients.

**Results:**

Twenty participants completed the study. Excellent intra-rater (intraclass correlation coefficient = 0.91 (extensor digitorum), 0.94 (anterior deltoid)) and good to excellent test–retest reliability (intraclass correlation coefficient = 0.69 (anterior deltoid), 0.84 (extensor digitorum)) was found for resting motor threshold. Minimal detectable change for resting motor threshold was found at 10.95% (extensor digitorum) and 16.35% (anterior deltoid) between first and third measurements. Motor-evoked potential amplitude of extensor digitorum communis had fair to good intra-rater (intraclass correlation coefficient = 0.50) and test–retest reliability (intraclass correlation coefficient = 0.65).

**Conclusions:**

Our results suggest that resting motor threshold is a reliable neurophysiological measure even for proximal shoulder muscles. Future research should further explore the reliability of motor-evoked potential amplitude before integration into neurological rehabilitation.

## Introduction

Transcranial magnetic stimulation (TMS), a non-invasive form of brain stimulation, can be used both as a corticomotor intervention and neurophysiological outcome measure. TMS allows the study of motor-evoked potentials (MEPs) resting thresholds and amplitudes of the upper limb as a measure of changes in corticomotor excitability of healthy people and people with neurological conditions when combined with electromyography (EMG).^1^ Upper limb activities such as reach to grasp require coordinated motor control of both proximal and distal musculature.^2^ Due to prominent cortical representations, investigation of the level of reliability of neurophysiological outcome measures has been more popular with distal than proximal muscles.^3–5^ However, the study of the psychometric properties of the outcome measure with proximal muscle areas is equivocal valuable.^6^

Reliability of a measure refers to the extent that the measurement is free from error and also consistent.^7^ Research exploring the reliability of TMS outcome measurements mainly involved the elbow, wrist and hand regions. Excellent test–retest reliability (intraclass correlation coefficient (ICC) = 0.92) has been found for resting motor threshold (RMT) for distal hand muscles; abductor pollicis brevis, first dorsal interosseus and abductor digiti minimi in young healthy adults but lower reliability for proximal muscles such as the biceps brachii (ICC = 0.75).^6,8^ Variable results have been presented about the reliability of MEP amplitude measures for proximal and distal muscles. Kamen^4^ demonstrated higher reliability for proximal muscles such as the biceps brachii than distal hand muscles; however, Brasil-Neto et al.^5^ and Corp et al.^9^ reported higher reliability for both in young adults. Interestingly, a higher intra-rater reliability (ICC = 0.67–0.99) has been reported for the abductor digiti minimi in older adults.^10^

None of the studies explored the reliability of TMS outcome measurements of the proximal shoulder region. In conditions such as stroke, overactivity of shoulder muscles contributes to reaching activities.^11^ Therefore, further analyses of the reliability of the outcome measure involving both proximal and distal muscles of an older healthy population warrant investigation. This study was part of a project where TMS measurement was also conducted with people with stroke. It was therefore useful to further investigate intra-rater, test–retest reliability and Minimal Detectable Change (MDC) of the RMT and amplitude of a more proximal shoulder muscle, anterior deltoid (AD), and compare it with new data from a distal wrist muscle, extensor digitorum communis (EDC) with an age-matched healthy population before carrying out this assessment with people with stroke.

## Methods

### Participants and recruitment

Healthy adults were recruited. Participants needed to be: (i) >18 years and (ii) able to provide informed consent. Participants were excluded if they had (i) impaired gross cognitive function; score of less than 24 of the Mini-Mental State Examination^12^; (ii) a neurological condition such as stroke; (iii) a history of epilepsy; (iv) previous brain neurosurgery; (v) had metal implants in the head including cochlear implants; (vi) been taking medications that influence cortical excitability; (vii) previous adverse effects with TMS and (viii) pregnancy. Participants were recruited through websites and participant databases of the University of Southampton.

### Setting and measurement procedure

Block randomization was used whereby each participant was randomized into either left cortical stimulation or right cortical stimulation. Two researchers (LTT and AED) carried out the experimental procedure at the Movement Laboratory of the Faculty of Health Sciences, University of Southampton. Demographic data including age and handedness were recorded. A TMS questionnaire was used to ensure that the participant fulfilled the criteria for TMS application.^13^

Surface EMG recording was then set-up to record the activity of the AD and ED muscles using the wireless portable Biometric EMG DataLog Bluetooth® system (Type number W4X8) equipment (Biometrics Ltd, Gwent, UK). Before attaching the bipolar electrodes, the skin was cleaned and wiped with an alcohol swab. The muscles on the participant’s arm were located according to the Seniam Guidelines.^14^ The AD was located by placing one finger width distal and anterior of the acromion. The electrode was orientated in the direction of the line between the acromion and the thumb. The ED was located by palpating the lateral epicondyle of the humerus and the styloid process of the radius and ulna and a mark was placed between the two points.^15^ Two SX230FW electrodes were placed on the marked muscle bellies of the left or right upper limb using a sticky pad. The reference electrode was placed around the wrist. The arm, flexed at the elbow, of the participant was placed on a pillow placed on the armrest of a wooden chair. Muscle activity signals with 1000 gain picked up by the electrodes were stored by the DataLog. The activity of the muscles was checked on the program during voluntary movement of the upper limb of the participant. The researcher monitored the activity of the arm muscles on the program and ensured that there was not any activity during the data collection procedure.

**Figure 1.**
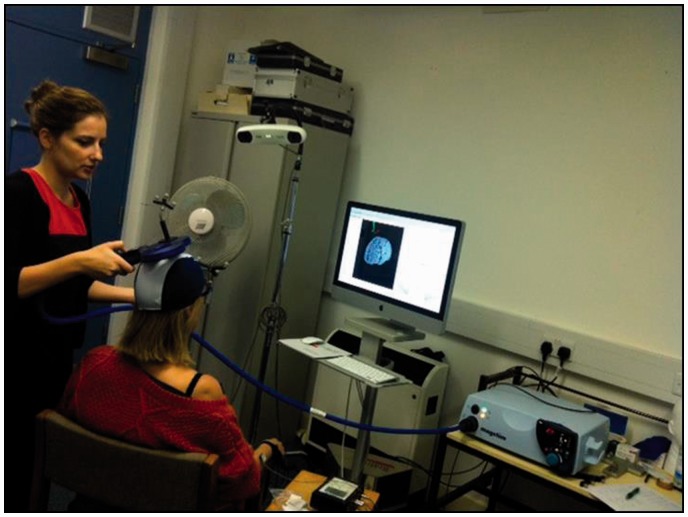
Experimental set-up of the TMS, Brainsight® and EMG equipment – the figure of eight magnetic coil was placed on the participant’s head to measure MEP responses of the AD and extensor digitorum muscles.

TMS was applied with a Magstim® 200^2^ device in combination with Brainsight® neuronavigation (Figure 1). A single pulse of magnetic stimulation was delivered to the motor region of the cortex (M1) of the right or left hemisphere by the figure-8-chaped coil at a 45° angle in a posterior–anterior plane, every 5–10 s until a MEP of the AD and ED muscles was noted on the EMG program. RMT was defined as the minimal TMS intensity to recruit a MEP >50 µV in at least five of 10 consecutive measurements in both muscles measured by EMG. The ‘hot spot’ locations were recorded on the standard MRI provided with the Brainsight® equipment. The MEPs were recorded from 100 to maximum 150% of RMT to measure the recruitment curves of AD and ED muscles at an interval of 5 s represented with a different colour.^9^ When AD MEP was elicited, in most cases it was seen in conjunction with ED MEP but with smaller amplitude.

Two measurements, with a 30 min rest between them, were carried out on one day (tests 1 and 2) and a third measurement three days later (test 3) by the same experimenter (LTT). To optimize accuracy, the TMS coil was placed in the same location of the participant’s head for all the measurements by using the Brainsight®. It was ensured that the same standardized protocol for EMG application was carried out for both sessions.

### Ethical approval

This study was approved by the University of Southampton Faculty of Health Sciences Ethics Committee (Ethics Number: 5382).

### Data and statistical analyses

Raw data from the DataLog was exported into MATLAB 2013b. Mean peak-to-peak amplitudes in millivolts of five MEPs of both muscles at 100–150% RMT on the three different occasions were calculated using a code written on MATLAB. For EDC, a MEP was elicited at a maximum of 130% in 17 participants and 150% in 15 participants. Therefore, the area of the Input/Output (I/O) curves of EDC from 100 to 130% and 100 to 150% was then calculated and analysed separately. The I/O curves could not be calculated for AD due to only a maximum of 120% could be reached in six participants. The reliability of RMT and MEP amplitudes was analysed by ICC using a two-way mixed model (Model 3,1) in SPSS Statistic 21. The interpretation for the ICC as described by Fleiss^16^ was used: 0.4 indicating poor, 0.4–0.75 indicating fair to good and 0.75 indicating excellent agreement. The coefficient of variation (CV) SD (pooled data test 1 and test 2) / Mean (pooled data test 1 and 2) x 100%, the standard error of measurement (SEM) (SEM = standard deviation from the first test × ($\sqrt{1-\mathrm{ICC}}$) and the minimal detectable change (MDC) (MDC = 1.96 × SEM × $\sqrt{2}$) for RMT of both muscles and MEP amplitude of EDC were also calculated.

## Results

Twenty participants (10 males, 10 females, mean age 58 years ± 11 SD, range 38–79 years) completed the study. Three participants were left handed and 17 were right handed. Two participants reported dizziness, headaches and discomfort at high TMS intensities which subsided after an hour.

The mean ED RMT at test 1 was 58.38% (SD 9.87), at test 2 was 59.29 % (SD 11.82) and at test 3 was 60.20% (SD 9.81). The mean AD RMT at test 1 was 77.17% (SD 10.59), at test 2 was 73.38% (SD 11.34) and at test 3 was 76.45% (SD 9.13). In all cases, the AD RMT was higher than the EDC RMT. MEPs of EDC were elicited in all participants and in 12 participants for AD. Excellent reliability was found between tests 1 and 2 (ICC = 0.91 (EDC), ICC = 0.94 (AD)) and excellent to good for tests 1 and 3 (ICC = 0.84 (EDC), ICC = 0.69 (AD)).

The area of I/O curves of MEP amplitude had fair to good reliability from 100 to 130% and 150% RMT between tests 1 and 2 (ICC = 0.50, 0.59) and tests 1 and 3 (ICC = 0.65, 0.69) (Table 1). CVs of the tests varied from 15 to 68%. MDCs of RMT were found at 7.18% for AD and 8.20% for EDC between tests 1 and 2 and 10.95% for EDC and 16.35% for ADC between tests 1 and 3. MDCs for EDC MEP amplitude ranged from 1.55 to 2.55 mV between tests 1 and 2 and 1.30–2.22 mV between tests 1 and 3 (Table 1).

**Table 1.** Mean (SD), ICC with 95% confidence intervals, SEM and MDC of resting motor threshold and motor-evoked potential area of anterior deltoid and extensor digitorum communis input/output curves between tests 1–2 and test 1–3.

	Means (SD)	ICC	95% CI	CV (%)	SEM	MDC %/mV
RMT (%MSO)	
Tests 1 and 2 (EDC)	58.38 (9.87) 59.29 (11.82)	0.91	0.75–0.95	18	2.96	8.20
Tests 1 and 3 (EDC)	58.38 (9.87) 60.20 (9.81)	0.84	0.64–0.93	33	3.95	10.95
Tests 1 and 2 (AD)	77.17 (10.59) 73.38 (11.34)	0.94	0.80–0.98	15	2.59	7.18
Tests 1 and 3 (AD)	77.17 (10.59) 76.45 (9.13) 76.81	0.69	0.15–0.91	26	5.90	16.35
EDC MEP area (mV) of I/O curves from 100 to 130%	
Tests 1 and 2	1.34 (0.80) 1.39 (1.04)	0.50	0.03–0.79	68	0.57	1.58
Tests 1 and 3	1.34 (0.80) 1.37 (0.66)	0.65	0.22–0.87	54	0.47	1.30
EDC MEP area of I/O curves from 100 to 150%	
Tests 1 and 2	2.44 (1.44) 2.44 (1.82)	0.59	0.06–0.86	67	0.92	2.55
Tests 1 and 3	2.44 (1.44) 2.56 (1.44)	0.69	0.24–0.89	58	0.80	2.22

AD: anterior deltoid; CI: confidence interval; CV: coefficient of variation; EDC: extensor digitorum communis; ICC: intraclass correlation coefficient; I/O: input/output; MDC: minimal detectable change; MEP: motor-evoked potential; mV: millivolt; MSO: Maximum Stimulator Output; RMT: resting motor threshold at maximum stimulator output; SEM: standard error of mean.

## Discussion

This was the first study to explore reliability of MEP RMT and amplitude of both upper limb proximal and distal muscles and it was identified that RMT is a more reliable measure even for a shoulder muscle. As was identified in previous research involving the biceps brachii, RMT does seem to be more reliable for distal ED than for the more proximal AD.^6^

Compared to previous research, lower reliability was found for measurement of MEP amplitude on proximal and distal muscles.^5,9^ This could have been due to an older population included compared to previous research. Additionally, a larger number of TMS stimuli were applied than the present study. It has been suggested that 10 instead of five TMS stimuli should be applied when investigating cortical excitability over multiple sessions of healthy adults.^17^ However, for proximal muscles such as AD, very high stimulation intensity was needed to elicit MEPs and therefore more stimuli could increase discomfort for participants.

Higher MDCs for RMT for both AD and EDC muscles were found compared to previous research where the majority included hand muscles.^18^ Before integrating such neurophysiological outcome measures in rehabilitation settings, further research should investigate MDC for both proximal and distal arm muscles. One must note that within the present study AD MEPs were elicited in 12 participants. This could be due to AD having a smaller cortical representation area than the EDC. However, no difference between cortical size and areal representations has been found when eliciting MEPs of proximal and distal muscles in young adults.^19^ Therefore, the older age of the participants could have been a contributing factor.

Temporal stability of an outcome measure is essential for use in clinical trials and clinical practice at multiple timepoints.^20^ MEP measurement is regularly used as a prognostic tool for upper limb recovery following stroke.^21^ It has been identified that when elicited MEPs and the ipsilesional RMT can be detected by 70% in the acute stage, upper limb motor impairment can also be resolved by 70% at 12 weeks’ post-stroke.^22^ Therefore, integrating such outcome measures in stroke rehabilitation could give an indication of which patients are most likely to improve their upper limb impairments. However, one must note that predictive algorithms focus on distal rather than both proximal and distal upper limb muscles.

There are a few limitations related to the present research. The study had a small sample size and participants were not selected randomly from the general population and therefore, the data cannot be generalized to all healthy young and old adults. The large CV results for MEP amplitude data, indicating high variability, were found and therefore that data should be considered with caution. The validity and the inter-rater reliability psychometric properties of RMT and MEP amplitude measurement were not examined in this study and therefore could not be explored. In addition, less data were obtained for the AD muscle. Having chosen a larger proximal muscle such as the middle deltoid muscle could have resulted in more MEP data.

Future research should explore the reliability of active in addition to RMT for proximal muscles. Moreover, for MEP amplitude to be used as an outcome measure for motor recovery and changes in cortical excitability, future research should explore if the presented results are applicable to people with neurological conditions.
